# Glabridin Therapy Reduces Chronic Allodynia, Spinal Microgliosis, and Dendritic Spine Generation by Inhibiting Fractalkine-CX3CR1 Signaling in a Mouse Model of Tibial Fractures

**DOI:** 10.3390/brainsci13050739

**Published:** 2023-04-29

**Authors:** Juan Long, Hongbing Liu, Zhimin Qiu, Zhong Xiao, Zhongqiu Lu

**Affiliations:** 1 Emergency Department, The First Affiliated Hospital of Wenzhou Medical University, Wenzhou 325000, China; 2 Wenzhou Key Laboratory of Emergency and Disaster Medicine, Wenzhou 325000, China; 3 Intensive Care Unit, Shaoxing People’s Hospital, Shaoxing 312000, China

**Keywords:** bone fracture, chemokine, chronic allodynia, fractalkine, glabridin, microglia activation, spine morphogenesis

## Abstract

Patients undergoing bone fractures frequently suffer from irritating chronic pain after orthopedic repairs. Chemokine-mediated interactions between neurons and microglia are important steps for neuroinflammation and excitatory synaptic plasticity during the spinal transmission of pathological pain. Recently, glabridin, the main bioactive component of licorice, has been shown to exhibit anti-nociceptive and neuroprotective properties for inflammatory pain. This present study evaluated the therapeutic potential of glabridin and its analgesic mechanisms using a mouse model of tibial fracture-associated chronic pain. Repetitive injections of glabridin were delivered spinally daily for 4 continuous days from days 3 to 6 after the fractures. Herein, we discovered that repeated administrations of glabridin (10 and 50 μg, but not 1 μg) could prevent prolonged cold allodynia and mechanical allodynia following bone fractures. A single intrathecal intervention with glabridin (50 μg) relieved an existing chronic allodynia two weeks following the fracture surgeries. Systemic therapies with glabridin (intraperitoneal; 50 mg kg^−1^) were protective against long-lasting allodynia caused by fractures. Furthermore, glabridin restricted the fracture-caused spinal overexpressions of the chemokine fractalkine and its receptor CX3CR1, as well as the elevated number of microglial cells and dendritic spines. Strikingly, glabridin induced the inhibition of pain behaviors, microgliosis, and spine generation, which were abolished with the co-administration of exogenous fractalkine. Meanwhile, the exogenous fractalkine-evoked acute pain was compensated after microglia inhibition. Additionally, spinal neutralization of fractalkine/CX3CR1 signaling alleviated the intensity of postoperative allodynia after tibial fractures. These key findings identify that glabridin therapies confer protection against inducing and sustaining fracture-elicited chronic allodynia by suppressing fractalkine/CX3CR1-dependent spinal microgliosis and spine morphogenesis, suggesting that glabridin is a promising candidate in the translational development of chronic fracture pain control.

## 1. Introduction

Due to the increasing number of construction injuries, road injuries, and industrial accidents, musculoskeletal traumas are clinically common and account for a huge strain on healthcare costs all over the world [1]. Patients undergoing bone fractures often experience chronic pain after orthopedic surgeries, for which there is no efficacious treatment [2,3]. Moreover, fracture-associated chronic pain is capable of inducing depression, cognitive impairment, and disability [4,5]. The accumulating literature emphasizes that neuroinflammation and excitatory synaptic plasticity drive central nociceptive sensitization, which is important in spinal perception of pro-nociception with different etiologies, such as tissue inflammation, nerve trauma, chemotherapies, and fractures [6,7,8]. However, the detailed molecular mechanisms underlying fracture-caused persistent pain were previously unknown.

Chemokines binding to their receptors contribute to the crosstalk between neurons and microglial cells in mediating neuronal plasticity and microglia activation in neuroinflammation-dependent pain neural circuitry that, subsequently, sustains the nociceptive phenotypes [9,10,11]. Fractalkine, termed chemokine CX3C motif ligand 1 (CX3CL1), which is exclusively expressed from spinal nociceptive neuronal cells, is emerging as a cardinal modulator of excitatory synaptic transmission and microgliosis via acting on its sole receptor CX3CR1 on microglial cells in the neuropathogenesis of opioid-induced hyperalgesia [12], myofascial low back pain [13], mechanical allodynia after tetanic stimulation of the sciatic nerve [14], and chronic pain induced by Leishmania amazonensis infections [15]. In addition, neuroinflammatory responses are gradually recognized as critical regulators for spine structural alternations and proliferations, resulting in the functional enhancement of pro-nociceptive sensory synapses in generating and maintaining chronic fracture pain [8,16]. Nevertheless, whether the spinal fractalkine/CX3CR1 cascades result in fracture-associated chronic pain, microglia activation, and spine morphogenesis is basically unexplored.

Several pharmacological approaches have been used to alleviate musculoskeletal pain in clinics, but prolonged utilization of these agents, including NSAIDs, corticosteroids, and opioids, may cause undesirable side effects [3,17,18]. On account of this, treatments using effective ingredients originated from plant extractions are of particular interest due to both improved medical obedience by patients and comparatively few side reactions. Licorice (*Glycyrrhiza glabra*) has been generally utilized in herbal medicine as a treatment for pulmonary, hepatic, and renal diseases, infections, arthritis, and even cancer, which is attributed to its numerous bioactive ingredients [19,20]. Of note, glabridin, the primary bioactive phytochemical isoflavone from licorice root, has been reported to have the therapeutic properties of inhibiting inflammatory responses and oxidation insults, as well as providing neuroprotection in different neurological conditions [21,22]. Recently, glabridin has been identified as valid in reducing acute inflammatory pain after intraplantar injections of capsaicin, formalin, and carrageenan for suppressing inflammation in rodent animals [23]. Nevertheless, up to now, there has been insufficient data regarding whether glabridin is protective against fracture-associated chronic pain.

This pre-clinical research evaluated the therapeutic properties of intrathecal (i.t.) and intraperitoneal (i.p.) interventions with glabridin for chronic pain states in mice undergoing tibial fractures and orthopedic surgeries. The spinal concentrations of fractalkine and CX3CR1, dendritic spine generation, and microgliosis were also assessed to elucidate the neuropathology of fracture pain and the analgesic mechanisms of glabridin. Thus, the novelties of this present research are that we revealed the amelioration of prolonged fracture allodynia by glabridin strategies via inhibiting the spinal fractalkine/CX3CR1-dependent spine plasticity and microgliosis.

## 2. Materials and Methods

### 2.1. Animals

Adult C57BL/6J mice (males; 8–10 weeks old) were housed in plastic cages in an artificially regulated 12-hour light–dark environment with free access to food and water. The room temperature and humidity were kept stable for all of the experiments. The animal experiment procedure was carried out in accordance with the National Institutes of Health Guide for the Care and Use of Laboratory Animals and approved by the Laboratory Animal Ethics Committee of the First Affiliated Hospital of Wenzhou Medical University (Zhejiang, China).

### 2.2. Drugs and Administration

Glabridin (, G9548, Sigma-Aldrich, St. Louis, MO, USA) was diluted in 10% dimethyl sulfoxide (D2650, DMSO; Sigma-Aldrich) for the injections. Recombinant fractalkine (Sigma-Aldrich, F2302), microglia inhibitor minocycline (ab120661, Abcam, Cambridge, UK), a neutralizing antibody against fractalkine (AF472, anti-fractalkine; R&D Systems, Minneapolis, MN, USA), and a neutralizing antibody against CX3CR1 (AF5825, anti-CX3CR1; R&D Systems) were diluted in 10% DMSO for the injections. For the i.t. injections, the spinal cord puncture was performed with a 30 G needle between the L_4_ and L_5_ levels to deliver the drugs (5 µL) to the cerebral spinal fluid under brief anesthesia with sevoflurane [24].

### 2.3. Mouse Model of Chronic Fracture Pain

The tibial fracture-elicited chronic nociceptive model was established as in previous reports [16,25]. Specifically, under sevoflurane (induction, 3.0%; surgery, 1.5%) anesthesia, a muscle disassociation was performed after a short incision from the knee to the midshaft of the left tibia. After the osteotomy, a 0.38 mm stainless steel pin was inserted into the tibia‘s intramedullary canal, and the incision was sutured with 6-0 prolene. For the sham-operated animals, both the incision and the muscle disassociation were implemented similarly, but no tibial fractures or intramedullary pinning were carried out.

### 2.4. Behavioral Testing

To assess for mechanical allodynia, the mice were placed on an elevated metal mesh platform in boxes and allowed to acclimatize for 2 h before the examination. The paw withdrawal threshold (PWT) of the mice was measured using the ascending series of von Frey hairs (Stoelting, Wood Dale, IL, USA) with bending forces of 0.16, 0.4, 0.6, 1.0, and 2.0 g. Specifically, each trial started with 0.16 g of von Frey hair to stimulate the mid-plantar surface of the left hind paw. Licking or withdrawal during the 5 s stimulus was characterized as a positive response. The next lower force was applied when a positive response was detected; otherwise, the next higher force was applied, and, finally, the PWT was calculated using Dixon’s up-and-down method [16,26].

To assess for cold allodynia, two acetone applications (20 μL each) were gently applied to the left hind paw bottom using a pipette, and the responses to the acetone were scored as follows: 0, no response; 1, quick withdrawal, paw stamping, or flicking; 2, prolonged withdrawal or repeated flicking of the paw; 3, repeated paw flicking and licking [16,25].

For the mice with tibial fractures and drug pre-administrations (such as glabridin, anti-fractalkine, or anti-fractalkine), the pain behavior experiments were conducted on days 7, 10, 14, and 21, respectively, following the orthopedic repairs. For the mice undergoing fractures and drug post-administrations (such as glabridin, anti-fractalkine, or anti-fractalkine), the pain behaviors were evaluated at 1, 3, 5, and 24 hours, respectively, following the acute exposure to drugs. For the mice receiving fractalkine injections, the pain behaviors were evaluated at 3, 6, and 12 hours, respectively, following the fractalkine applications.

### 2.5. ELISA Analysis

An enzyme-linked immunosorbent assay (ELISA) was utilized for measuring the concentrations of fractalkine (ab100683, Abcam), CX3CR1 (SAB3500204; Sigma-Aldrich), and Iba-1 (SAB2500042; Sigma-Aldrich) in the L_4–5_ segments of the spinal cord. The spinal cord tissues were homogenized into a lysis buffer with phosphatase inhibitors and proteases. A BCA protein assay (Pierce) was used for determining the concentrations of the proteins. Each reaction was performed in a 96-well plate and 100 μg of the protein samples were used. All of the ELISA experiments followed the manufacturer’s protocol. The levels of fractalkine, CX3CR1, and Iba-1 were normalized to the total protein amounts and calculated using standard curves.

### 2.6. Golgi Staining

As described previously [26,27], the freshly dissected spinal dorsal horns were immersed in a Golgi-Cox solution for 14 days at 25 °C. Sections (100 μm) were placed on 2% gelatinized microscope slides, rinsed with distilled water for 60 s, soaked in ammonium hydroxide for 30 min in the dark, rinsed with distilled water for 60 s, and placed in a Kodak Fix for image capture for 30 min. The images were acquired with an Olympus Eclipse 80i microscope (Olympus, Tokyo, Japan). For the quantification, three sections were analyzed for each animal. To examine the morphology of the dendritic spines, each individual spine was traced and counted manually.

### 2.7. Immunofluorescence

Under deep anesthesia, the mice were transcardially perfused with pre-cooled PBS followed by 4% paraformaldehyde. The spinal cord was blown out using the hydraulic pressure method. The L_4_-L_5_ spinal cord was dissected and dehydrated in 30% sucrose for 2 days. The tissues were then frozen in O.C.T. and cut into 8 µm frozen sections using a cryostat (Leica Biosystems, Wetzlar, Germany). The sections were blocked with 0.3% Triton X-100 for 10 min and 5% goat serum for 1 h. They were then incubated with the primary antibody anti-Iba-1 (1:200; ab178847, Abcam) overnight at 4 °C. After rinsing three times with PBS, the sections were washed three times with PBS and incubated with a fluorescence-labeled secondary antibody for 60 min. The images were collected using a fluorescence microscope (Olympus, Tokyo, Japan).

### 2.8. Statistical Analysis

GraphPad Prism 8 (GraphPad Software, San Diego, CA, USA) was used for all of the statistical analyses. All data were presented as means (standard error of the mean, SEM). The differences between the groups were compared using a one-way or two-way analysis of variance (ANOVA) followed by Bonferroni post hoc tests. The criterion for statistical significance was *p* < 0.05.

## 3. Results

### 3.1. Intrathecal Pre-Administration of Glabridin Prevents Tibial Fracture-Associated Chronic Allodynia following Orthopedic Surgeries

**Figure 1:**
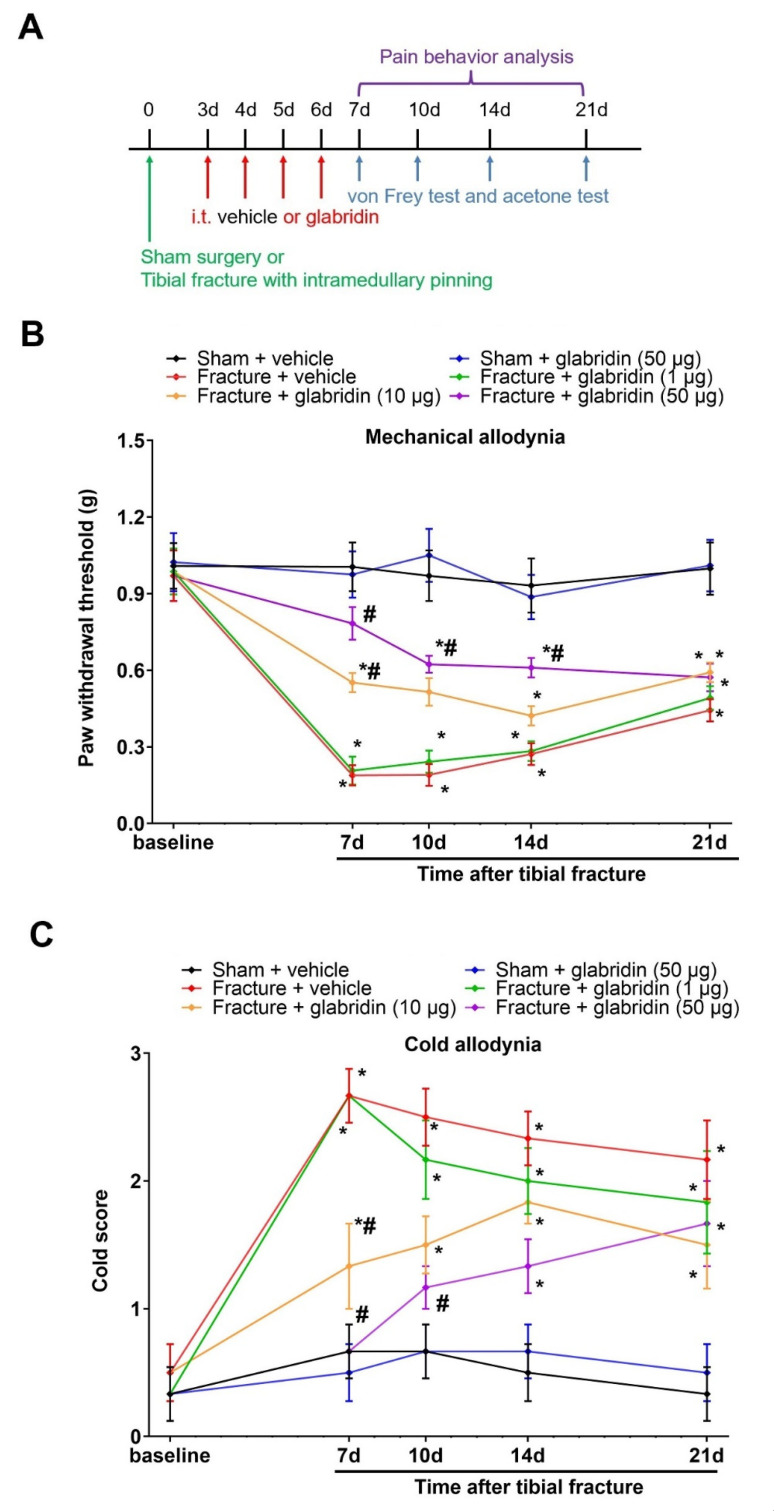
Pretreatment with glabridin via the intrathecal route decreases the initiation of fracture-associated allodynia. (**A**) Experimental designs to explore the preventive properties of glabridin on prolonged pro-nociception in a mouse model of tibial fractures. The behavioral phenotypes of mechanical allodynia (**B**) and cold allodynia (**C**) were observed. All behavioral results are shown as means ± SEM (*n* = 6) and analyzed by a two-way ANOVA with Bonferroni post hoc comparisons. * *p* < 0.05 vs. group sham + vehicle; # *p* < 0.05 vs. group fracture + vehicle; i.t., intrathecal.

First, no differences in the basal PWT and cold response to acetone were detected between the groups (*p* > 0.05; *n* = 6; Figure 1A–C). After the tibial fractures, the von Frey experiments found that mechanical allodynia was observed on day 7, peaked on day 14, and was sustained for at least 21 days in the ipsilateral hind paw, as identified by the robust reduction in the mechanical PWT in comparison with the sham surgeries (*p* < 0.05; *n* = 6; Figure 1B). Similarly, in the acetone experiments, as compared to the sham animals, the fractures of the orthopedic surgeries caused long-lasting cold allodynia (>21 d), as represented by the persistent hyper-response to acetone (*p* < 0.05; *n* = 6; Figure 1C).

To evaluate the therapeutic potential of glabridin for chronic pain caused by fractures (mechanical allodynia and cold allodynia), i.t. injections of glabridin (1, 10, and 50 μg) were carried out daily for 4 consecutive days on days 3, 4, 5, and 6 (in the early phase) in mice subjected to tibial fractures from orthopedic surgeries (Figure 1A). Strikingly, the central (spinal) therapies of glabridin at the doses of 10 μg and 50 μg, but not 1 μg, prevented fracture-elicited chronic pain, as demonstrated by the considerable elevation in the mechanical PWT (F(5, 150) = 57.79; *p* < 0.0001; *n* = 6, two-way ANOVA; Figure 1B) and the abrupt decrease in the cold response scores (F(5, 150) = 34.22; *p* < 0.0001; *n* = 6; two-way ANOVA; Figure 1C). Moreover, this attenuation of allodynia by the glabridin-dependent dosage lasted for 1–7 days.

### 3.2. Intrathecal Glabridin Reduces Spinal Fractalkine/CX3CR1 Expression and Microgliosis following Tibial Fractures and Orthopedic Surgeries

**Figure 2:**
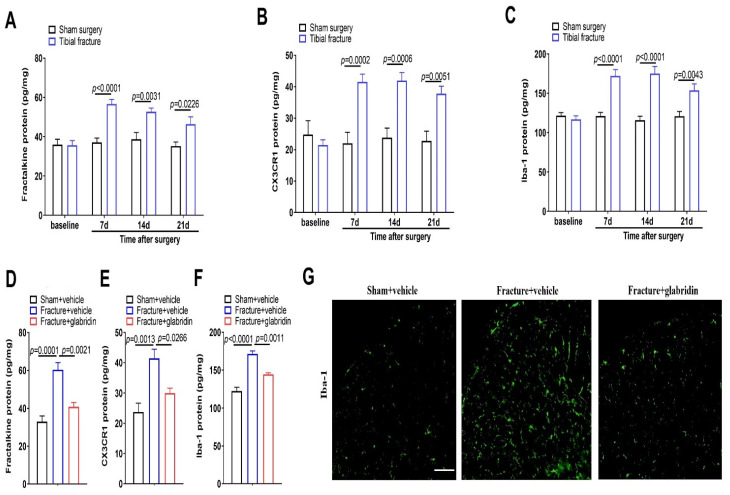
The fractures induce a spinal increase in fractalkine and CX3CR1, and microgliosis is reduced following repetitive injections with glabridin. (**A**–**C**) The ELISA experiments detected the dynamic alternations of spinal fractalkine, CX3CR1, and Iba-1 proteins following the fractures and orthopedic surgeries (*n* = 5). Intrathecal glabridin (50 μg) was delivered daily for 4 consecutive days on days 3, 4, 5, and 6 following the fractures. (**D**–**F**) The ELISA experiments revealed that the up-modulation of spinal fractalkine, CX3CR1, and Iba-1 proteins after the fractures and orthopedic surgeries was inhibited by the glabridin pretreatment (*n* = 5). (**G**) The immunofluorescence staining showed representative photomicrographs of Iba-1 in the dorsal horn following the fractures and glabridin exposure (the scale bar is 50 μm). All data are expressed as means ± SEM and analyzed by a one-way or two-way ANOVA with Bonferroni post hoc comparisons.

Chemokines and their receptors in the dorsal horn mediate microglia activation and neuroinflammatory responses, which is an essential hallmark of chronic allodynia after nerve injuries and bone cancer [9,10]. Herein, in the ELISA experiments, as compared to the sham operations, the spinal up-modulations of fractalkine, CX3CR1, and Iba-1 (a marker of microglia) expressions occurred on day 7, peaked on day 14, and lasted for more than 21 days in the animals subjected to fractures (*p* < 0.05; *n* = 5; Figure 2A–C), which was consistent with the time course of persistent allodynia behaviors. Together, these suggest that tibial fractures with orthopedic repairs induce long-lasting overexpressions of fractalkine and CX3CR1, as well as microgliosis in the dorsal horn, which is implicated in the neuropathophysiological process of chronic allodynia.

Furthermore, we examined the spinal levels of fractalkine, CX3CR1, and Iba-1 to determine if they may be required for the anti-nociceptive effects of glabridin in the fractured animals. Noteworthy, as compared to the fractures, pretreatment with glabridin (50 μg) reduced the spinal overexpressions of fractalkine (F(2, 12) = 21.34; *p* = 0.0001; *n* = 5; one-way ANOVA; Figure 2D), CX3CR1 (F(2, 12) = 11.93; *p* = 0.0014; *n* = 5; one-way ANOVA; Figure 2E), and Iba-1 (F(2, 12) = 39.31; *p* < 0.0001; *n* = 5; one-way ANOVA; Figure 2F). Additionally, the immunofluorescence experiments revealed that tibial fracture-mediated microgliosis was abrogated following the glabridin interventions (Figure 2G). All of these data imply that glabridin therapies prevent chronic allodynia through spinal suppression of neuroinflammation.

### 3.3. Post-Treatment with Glabridin via Intrathecal Route Relieved the Existing Prolonged Allodynia following Orthopedic Surgeries with Tibial Fractures

**Figure 3:**
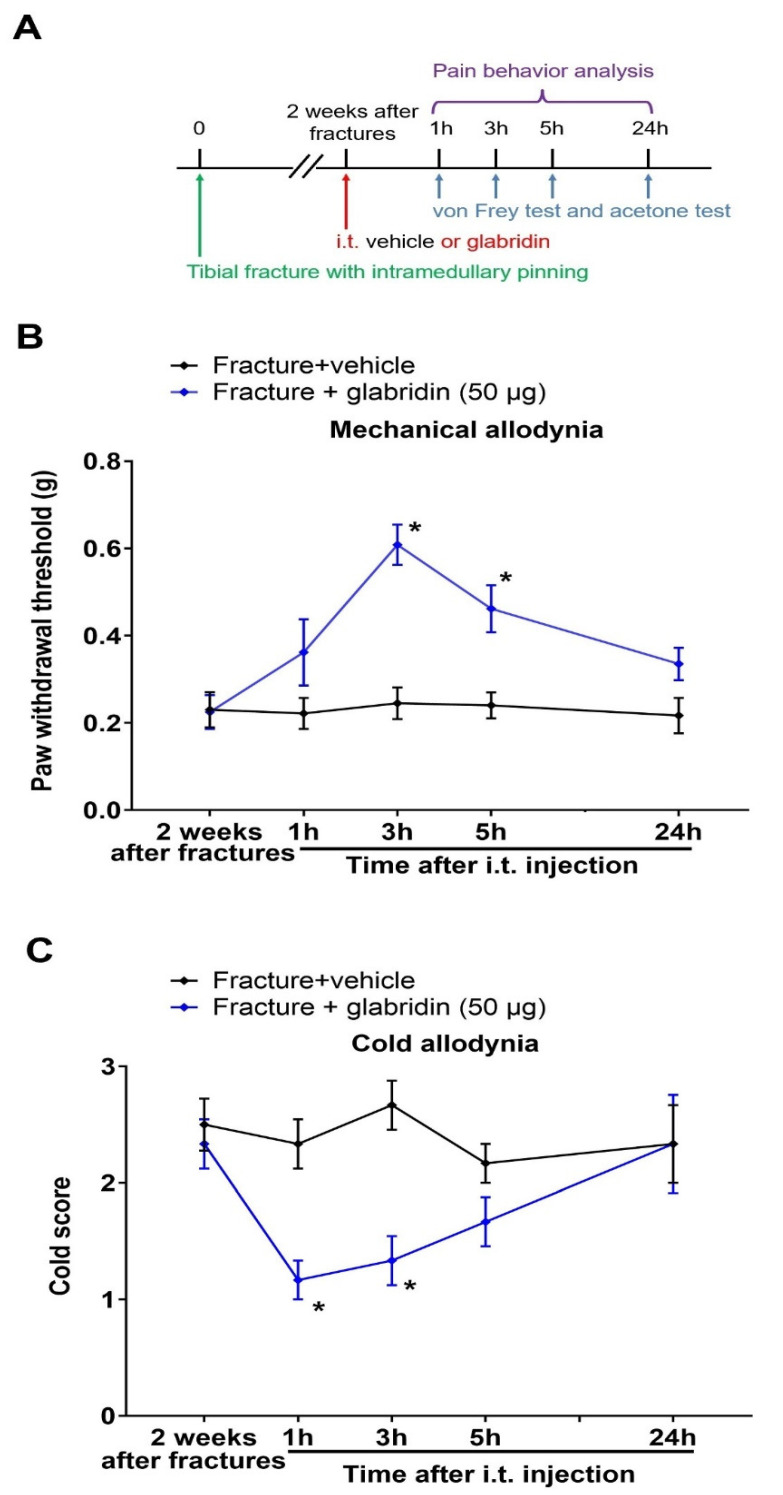
Post-treatment with glabridin via the intrathecal route attenuates the existing prolonged allodynia caused by the tibial fractures. (**A**) Experimental designs to explore the therapeutic properties of glabridin on the established persistent allodynia in the mouse model of tibial fractures. The behavioral phenotypes of mechanical allodynia (**B**) and cold allodynia (**C**) were recorded. Results are shown as means ± SEM (*n* = 6) and analyzed by a two-way ANOVA with Bonferroni post hoc comparisons. * *p* < 0.05 vs. group fracture + vehicle; i.t., intrathecal.

Once the reduction in the initiation of allodynia by the pre-administration of glabridin was verified, we further investigated the potential role of i.t. post-treatment with glabridin in the existing chronic allodynia (Figure 3A). In the von Frey experiments (Figure 3B), a single exposure to glabridin (50 μg) two weeks after the fractures (in the late phase) exhibited a short-term alleviation in the existing mechanical allodynia, as characterized by the considerable elevation in the PWT at 3 h (*p* < 0.0001) and 5 h (*p* = 0.0054) post-injection in the mice subjected to the fractures. Simultaneously, in the acetone experiments (Figure 3C), the post-treatment with glabridin restrained the existing cold allodynia, as manifested by the remarkable decrease in the cold response to acetone at 1 h (*p* = 0.0084) and 3 h (*p* = 0.002) post-injection.

### 3.4. Spinal Suppression of Fractalkine/CX3CR1 Signaling Protects against Tibial Fracture-Associated Chronic Allodynia

**Figure 4:**
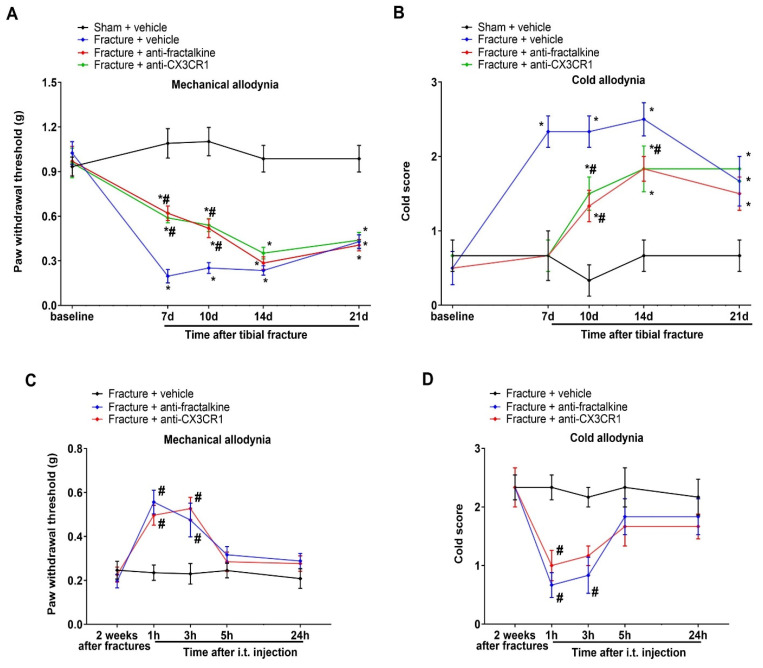
Pharmacological inhibition of fractalkine/CX3CR1 protects against fracture-associated chronic allodynia. (**A**,**B**) The anti-fractalkine and anti-CX3CR1 were intrathecally applied (20 μg) on days 3, 4, 5, and 6 after the fracture surgeries. The mechanical allodynia and cold allodynia were measured. (**C**,**D**) Post-treatment with anti-fractalkine and anti-CX3CR1 (20 μg) via the intrathecal route two weeks following the orthopedic surgeries considerably ameliorated the existing mechanical and cold allodynia. All behavioral results are shown as means ± SEM (*n* = 6) and analyzed by a two-way ANOVA with Bonferroni post hoc comparisons. * *p* < 0.05 vs. group sham + vehicle; # *p* < 0.05 vs. group fracture + vehicle.

Next, to test if the fractalkine cascades in the spinal dorsal horns are important in generating chronic allodynia, repetitive anti-fractalkine (i.t.; 20 μg) and anti-CX3CR1 (i.t.; 20 μg), respectively, were injected on a daily basis from days 3 to 6 following the tibial fractures. Interestingly, the pre-administration of anti-fractalkine and anti-CX3CR1 showed a prevention of mechanical and cold allodynia, which continued for 1–4 days, as reflected by the increase in the mechanical PWT (F(3, 100) = 75.89; *p* < 0.0001; *n* = 6; two-way ANOVA; Figure 4A) and the decrease in the cold scores (F(3, 100) = 23.86; *p* < 0.0001; *n* = 6; two-way ANOVA; Figure 4B) in the rodents subjected to the fractures. Furthermore, a single post-treatment with anti-fractalkine (i.t.; 20 μg) and anti-CX3CR1 (i.t.; 20 μg) two weeks following the tibial fractures ameliorated the existing mechanical allodynia (F(2, 75) = 14.73; *p* < 0.0001; *n* = 6; two-way ANOVA; Figure 4C) and cold allodynia (F(2, 75) = 12.82; *p* < 0.0001; *n* = 6; two-way ANOVA; Figure 4D). Collectively, these findings identified that the spinal activation of fractalkine-CX3CR1 signaling is sufficient to initiate and sustain chronic allodynia caused by tibial fractures from orthopedic repairs.

### 3.5. Recombinant Fractalkine Abolished Glabridin-Induced Anti-Nociception, Spinal Inhibition of CX3CR1 Expression, and Microgliosis

**Figure 5:**
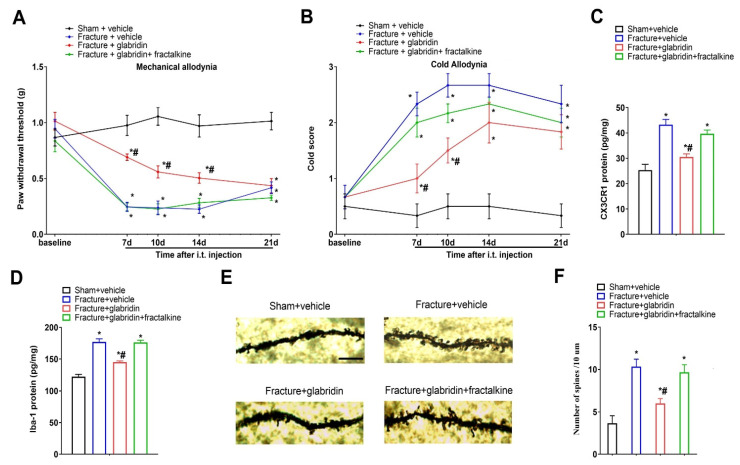
Spinal exposure to exogenous fractalkine abolishes glabridin-induced anti-nociceptive effects on chronic allodynia. Glabridin (50 μg) and recombinant fractalkine (50 ng) were intrathecally injected on days 3, 4, 5, and 6 after the fracture surgeries. The behavioral phenotypes of mechanical allodynia (**A**) and cold allodynia (**B**) were recorded. (**C**,**D**) The ELISA experiments identified changes in the spinal CX3CR1 and Iba-1 proteins after the fractures and the glabridin and fractalkine interventions. (**E**,**F**) Representative photomicrographs and statistical results of the spine morphology following the fractures and glabridin and fractalkine treatments (the scale bar is 5 μm). All behavioral results (*n* = 6) and biochemical results (*n* = 3–5) are shown as means ± SEM and analyzed by a one-way or two-way ANOVA with Bonferroni post hoc comparisons. * *p* < 0.05 vs. group sham + vehicle; # *p* < 0.05 vs. group fracture + vehicle.

Next, we evaluated if the spinal fractalkine pathway is involved in the analgesic mechanisms of glabridin. Intriguingly, the co-intervention with recombinant fractalkine (i.t.; 50 ng) abrogated the prevention of mechanical allodynia (F(3, 100) = 89.7; *p* < 0.0001; *n* = 6; two-way ANOVA; Figure 5A) and cold allodynia (F(3, 100) = 44.59; *p* < 0.0001; *n* = 6; two-way ANOVA; Figure 5B) by the glabridin pretreatment (i.t.; 50 μg) in the mouse model of tibial fractures. In parallel, the spinal exposure to recombinant fractalkine reversed the glabridin-induced reductions of CX3CR1 (F(3, 16) = 21.36; *p* < 0.0001; *n* = 5; one-way ANOVA; Figure 5C) and Iba-1 (F(3, 16) = 51.46; *p* < 0.0001; *n* = 5; one-way ANOVA; Figure 5D) expressions in the dorsal horn of the rodents undergoing bone fractures. These results provide strong evidence that fractalkine/CX3CR1-dependent microgliosis is a therapeutic target of glabridin anti-nociception in pro-nociception states.

### 3.6. Glabridin Reduces Fracture-Induced Spinal Dendritic Spine Generation, Which Is Compensated by Co-Administration of Recombinant Fractalkine

Given that the previous literature emphasized the key contributions of dendritic spine morphogenesis to functional neuroplasticity in the neuropathogenesis of fracture-elicited chronic allodynia and opioid-induced hyperalgesia [16,26,27], we next evaluated the substantial effects of glabridin on dendritic spine morphology. Intriguingly, the repetitive pretreatments with glabridin (i.t.; 50 μg) suppressed the generation of dendritic spines due to tibial fractures with pin insertions (*p* = 0.0404; Figure 5E,F). More importantly, the co-administration of recombinant fractalkine (i.t.; 50 ng) abolished the glabridin-induced down-modulation of spine production (F(3, 8) = 10.12; *p* = 0.0042; *n* = 3; one-way ANOVA; Figure 5E,F). These results revealed a previously unrecognized interaction between fractalkine cascades and dendritic spine morphogenesis in spinal pain transduction and glabridin analgesia.

### 3.7. Intrathecal Injection of Recombinant Fractalkine Evokes Acute Allodynia, Which Is Compensated by Co-Administration of Minocycline

**Figure 6:**
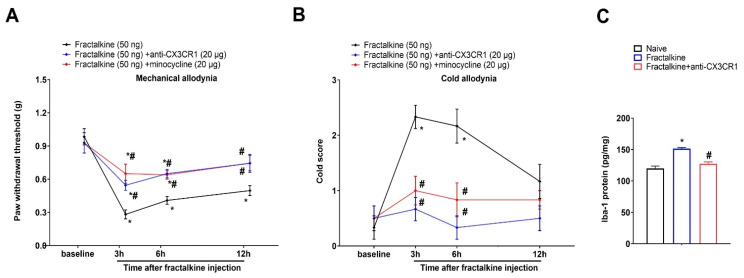
Acute nociceptive phenotypes are initiated by exogenous fractalkine and ameliorated by co-applications of minocycline. The microglial inhibitor minocycline (i.t.; 20 μg) and anti-CX3CR1 (i.t.; 20 μg) were given one hour prior to the delivery of the recombinant fractalkine (i.t.; 50 ng). (**A**,**B**) The exogenous fractalkine-evoked acute nociceptive phenotypes were ameliorated following the pre-applications of minocycline and anti-CX3CR1, respectively. All behavioral results are shown as means ± SEM (*n* = 6) and analyzed by a two-way ANOVA with Bonferroni post hoc comparisons. * *p* < 0.05 vs. baseline; # *p* < 0.05 vs. group recombinant fractalkine (50 ng). The biochemical experiments were performed 3 hours following the fractalkine injections. (**C**) The ELISA experiments showed that anti-CX3CR1 decreased the overexpression of the Iba-1 proteins in the dorsal horn following the exogenous fractalkine injections. All biochemical results are expressed as means ± SEM (*n* = 5) and analyzed by a one-way ANOVA with Bonferroni post hoc comparisons. * *p* < 0.05 vs. naïve; # *p* < 0.05 vs. group recombinant fractalkine.

Next, we further explored whether spinal microglia inhibition might block fractalkine-dependent pro-nociceptive sensitization. Herein, the recombinant fractalkine administration (i.t.; 50 ng) elicited an acute and transient allodynia phenotype. Then, anti-CX3CR1 (i.t.; 20 μg) or minocycline (i.t.; 20 μg) was given 30 min prior to the delivery of the exogenous fractalkine. We reported that the co-administration of anti-CX3CR1 prevented the decrease in the mechanical PWT (F(2, 60) = 11.07; *p* < 0.001; *n* = 6; two-way ANOVA; Figure 6A) and the increase in the cold response scores (F(2, 60) = 17.99; *p* < 0.001; *n* = 6; two-way ANOVA; Figure 6B) following the fractalkine exposure, implying that exogenous fractalkine-associated acute allodynia might be CX3CR1 activity-dependent. Furthermore, the fractalkine-caused overexpression of spinal Iba-1 at 3 h post-injection was compromised by CX3CR1 neutralization (F(2, 12) = 28.96; *p* < 0.001; *n* = 5; one-way ANOVA; Figure 6C). In parallel, the pretreatment with minocycline restrained the fractalkine-elicited mechanical allodynia (Figure 6A) and cold allodynia (Figure 6B). Overall, these specific results further summarized the cardinal link between fractalkine/CX3CR1 and microgliosis in spinal neuroinflammation and pain perception.

### 3.8. Systemic Therapy of Glabridin Ameliorates Fracture-Associated Chronic Allodynia Behaviors following Orthopedic Surgeries

**Figure 7:**
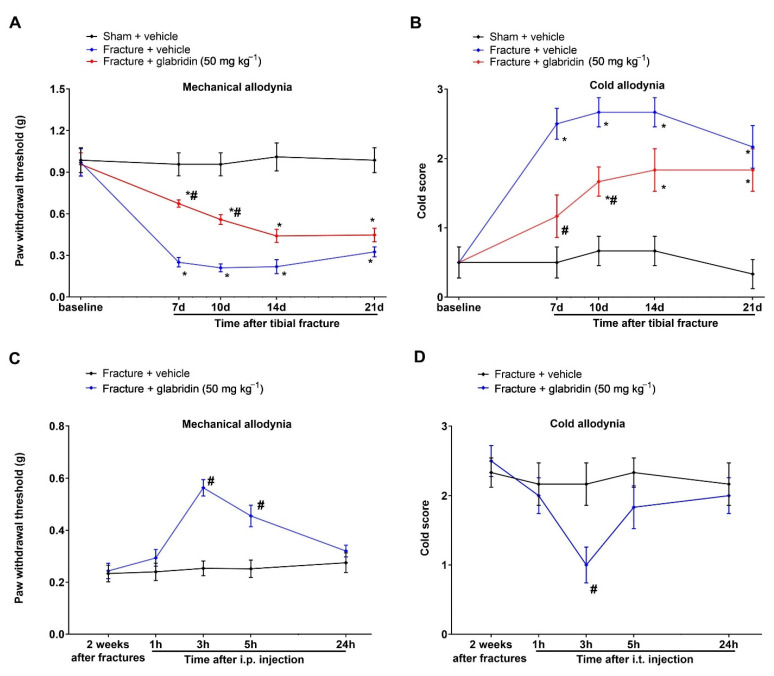
Intraperitoneal injections of glabridin reduce the generation and maintenance of chronic allodynia elicited by the tibial fractures. (**A**,**B**) Glabridin was intraperitoneally injected (50 mg kg^−1^) on days 3, 4, 5, and 6 following the fracture surgeries. The mechanical and cold pro-nociceptive phenotypes were measured. (**C**,**D**) The intraperitoneal application of glabridin (50 mg kg^−1^) two weeks following the fractures considerably ameliorated the existing mechanical and cold allodynia. All behavioral results are shown as means ± SEM (*n* = 6) and analyzed by a two-way ANOVA with Bonferroni post hoc comparisons. * *p* < 0.05 vs. group sham + vehicle; # *p* < 0.05 vs. group fracture + vehicle.

Finally, we explored if the systemic therapy of glabridin was also valid in controlling chronic fracture allodynia. Therefore, we administered glabridin (50 mg kg^−1^) following the intraperitoneal injections once a day from days 3 to 6 for 4 continuous days following the tibial fractures from the orthopedic surgeries. As expected, the pre-administration of glabridin reduced the initiation of mechanical and cold allodynia, as reflected by the increase in the PWT (F(2, 75) = 95.06; *p* < 0.0001; *n* = 6; two-way ANOVA; Figure 7A) and the decrease in the cold scores (F(2, 75) = 51.65; *p* < 0.0001; *n* = 6; two-way ANOVA; Figure 7B) in the fracture-treated rodents. Moreover, post-treatment with intraperitoneal glabridin (50 mg kg^−1^) was given two weeks following the orthopedic surgeries. A significant inhibitory effect on the existing mechanical allodynia was observed 3 h (*p* < 0.0001; Figure 7C) and 5 h (*p* = 0.0003; Figure 7C) after a single exposure to glabridin. In addition, the systemic post-treatment with glabridin showed a transient recovery in the increased cold response to acetone 3 h after a single injection (F(1, 50) = 4.69; *p* = 0.035; *n* = 6; two-way ANOVA; Figure 7D).

## 4. Discussion

Licorice has a long history as a medical drug for humans in treating hepatitis, gastrointestinal disorders, and cancer, while, recently, it has also exhibited analgesic roles in muscular pain, joint pain, and chemotherapy-induced neuropathic pain [19,20,28]. However, no literature has mentioned whether glabridin, the main bioactive compound of licorice, has therapeutic potential for controlling fracture-associated pain. Herein, our pre-clinical research recapitulates the undetermined property of glabridin as an effective alleviator of persistent allodynia through the spinal suppression of fractalkine/CX3CR1-dependent microgliosis and dendritic spine morphogenesis using a mouse model of tibial fractures.

Recently, chemokines and their receptors have attracted much attention due to their emerging contribution to spinal transmissions of nociceptive signals [6,9,10]. A study by Wang and colleagues revealed the implication of the chemokine CC motif ligand 1 (CCL1) and its receptor CCR8 in neuroinflammation and neural plasticity in fracture-associated behavioral pain hypersensitivity [29]. Another study by Zhang and colleagues indicated that spinal inhibition of the chemokine CC motif ligand 21 (CCL21) pathway is effective against tibial fracture-associated chronic pain syndromes by blocking microgliosis and neuronal excitability [30]. Actually, microglia activation in the spinal dorsal horn is a pivotal hallmark of chronic fracture pain pathogenesis [5,8]. Given that spinal fractalkine is a central microglia-activating contributor to pathological nociceptive sensation [9], we investigated whether fractalkine signaling regulates chronic allodynia caused by fractures. Herein, we first found a persistent increase in the fractalkine/CX3CR1 levels and microgliosis in the spinal dorsal horns in rodents subjected to tibial fractures, matching the development of prolonged nociceptive behaviors. We also provided the first evidence that the spinal neutralization of fractalkine/CX3CR1 signaling prevents and relieves fracture-associated allodynia in the early and late phases. Furthermore, exogeneous fractalkine injections via the spinal route evoke acute allodynia and activate microglial cells in the animals, which are compromised by CX3CR1 inhibition. In parallel, the pharmacological blockage of microglia activation reduces the fractalkine-caused acute pain. These intriguing findings from the behavioral and biochemical experiments forcefully demonstrate the identification of the fractalkine/CX3CR1 cascade and microgliosis in the generation and maintenance of allodynia following fractures, implying that inhibiting these may offer an effective therapeutic target for the prolonged states of allodynia.

The current investigation, for the first time, indicates that repeated administrations of i.t. glabridin (10 and 50 μg, but not 1 μg) impair the initiation of tibial fracture-associated allodynia. We next uncover the attenuation of the existing prolonged fracture allodynia by a single delivery of glabridin (50 μg). Moreover, systemic (intraperitoneal) delivery of glabridin (50 mg kg^−1^) is enough to prevent and alleviate fracture-associated chronic allodynia. Additionally, this is the first evidence revealing that the pre-administration of glabridin restricts spinal fractalkine secretion, CX3CR1 accumulation, and microgliosis in fractured animals. Strikingly, the glabridin-mediated anti-nociception and spinal inhibition of CX3CR1 expression and microgliosis are completely compensated following the co-administration of exogenous fractalkine. Mechanistically, these findings from our detailed experiments manifest that glabridin therapies are protective against generating and sustaining fracture-elicited chronic allodynia by downregulating fractalkine-dependent CX3CR1 cascades and microgliosis in the spinal dorsal horn. Still, how glabridin modulates chemokine fractalkine signaling to inhibit spinal excitatory neuronal hyperexcitability needs further investigation.

Dendritic spine structural plasticity is critical for the functional alternations of nociceptive synapses in central sensitization during pathological pain conditions, including complete Freund’s adjuvant (CFA)-associated inflammatory pro-nociception, nerve trauma-evoked neuropathological allodynia, and remifentanil-induced postoperative hyperalgesia [27,31,32]. A recent study recapitulates that the pharmacological suppression of dendritic spine alternations is effective against fracture-caused behavioral allodynia [26]. Supporting this notion, our current study discovers a decrease in the spine generation in the neurons of the dorsal horns in the fractured animals following analgesic therapies with glabridin. However, how glabridin downregulates spine morphogenesis remains unclear. Another study by Cui and colleagues reports that neuroinflammatory responses lead to modifications of the spine’s structures and numbers in the development of post-surgical nociceptive syndromes caused by fractures [16]. Given the requirement of fractalkine/CX3CR1 for neuroinflammation and glabridin analgesia in spinal pain processing, we further evaluated if fractalkine signaling is involved in the structural alternations of the spine. As expected, we, for the first time, find that the glabridin-induced reduction in dendritic spine generation is abolished after exogenous fractalkine intervention. The above results identify that glabridin therapies attenuate fracture allodynia and restrict synaptogenesis and neuroplasticity via spinal modulation of the fractalkine pathway. However, the correlation between fractalkine/CX3CR1 and spine morphogenesis following fracture surgeries is largely undefined.

Actually, it is clarified that microgliosis involves the development of pathological pain by secreting proinflammatory contributors (such as TNF-α and IL-18) and neuropeptides/neurotrophins (such as BDNF and NGF), which facilitate the release of neurotransmitters from nociceptive afferents of the spinal dorsal horn to sensitize the excitatory nociceptive neuronal cells [9]. Consequently, it would be interesting to study if these chemical mediators are substantial downstream targets in the analgesic properties of glabridin therapies. Additionally, oxidative insults are capable of inducing synaptic plasticity in opioid-induced hyperalgesia, paclitaxel-induced peripheral neuropathic allodynia, and bone cancer pain [33,34]. Given the potent anti-oxidative properties of glabridin [20,35,36], further studies are warranted to determine if oxidative insults are also implicated in the analgesia of glabridin for controlling fracture allodynia.

There are several limitations to this study. First, we did not study the anti-allodynia potential of glabridin in female animals, which should be explained by further investigations. Second, drug or antibody administration via the intrathecal route can act on both the dorsal root ganglions and the spinal cord; thus, it is a concern if the fractalkine-CX3CR1 cascades in the dorsal root ganglion are implicated in the analgesia of glabridin therapies. Third, although recombinant fractalkine and neutralizing antibodies against fractalkine/CX3CR1 were employed to explain the analgesic mechanisms of glabridin, future studies should focus more on how to use genetic tools to knockdown and overexpress the fractalkine genes and proteins. Fourth, we did not compare glabridin with positive control drugs, which should be taken into account in the future. Apart from microglia activation, astrocyte activation also underlies spinal neuroinflammation, synaptogenesis, and central pain sensitization [37,38]. Thus, another weakness is the failure to reveal the potential role of astrocyte-dependent signaling in glabridin analgesia.

In summary, the current findings reveal for the first time that glabridin reduces the spinal overexpression of fractalkine, CX3CR1, and microgliosis, which is associated with the prevention and attenuation of allodynia behaviors. These results strongly imply that glabridin therapy and fractalkine/CX3CR1 inhibition are promising candidates in the translational development of chronic pain management.

## Data Availability

All data relevant to the study are included in the article as figures. Data are available from the corresponding author upon reasonable request.

## References

[1] Chen W., Lv H., Liu S., Liu B., Zhu Y., Chen X., Yang G., Liu L., Zhang T., Wang H. (2017). National incidence of traumatic fractures in China: A retrospective survey of 512,187 individuals. Lancet Glob. Health.

[2] Khan J.S., Devereaux P., LeManach Y., Busse J.W. (2016). Patient coping and expectations about recovery predict the development of chronic post-surgical pain after traumatic tibial fracture repair. Br. J. Anaesth..

[3] McVeigh L.G., Perugini A.J., Fehrenbacher J.C., White F.A., Kacena M.A. (2020). Assessment, Quantification, and Management of Fracture Pain: From Animals to the Clinic. Curr. Osteoporos. Rep..

[4] Tajerian M., Leu D., Zou Y., Sahbaie P., Li W., Khan H., Hsu V., Kingery W., Huang T.T., Becerra L. (2014). Brain Neuroplastic Changes Accompany Anxiety and Memory Deficits in a Model of Complex Regional Pain Syndrome. Anesthesiology.

[5] Zhang M.-D., Barde S., Yang T., Lei B., Eriksson L.I., Mathew J.P., Andreska T., Akassoglou K., Harkany T., Hökfelt T.G.M. (2016). Orthopedic surgery modulates neuropeptides and BDNF expression at the spinal and hippocampal levels. Proc. Natl. Acad. Sci. USA.

[6] Baral P., Udit S., Chiu I.M. (2019). Pain and immunity: Implications for host defence. Nat. Rev. Immunol..

[7] Li W.-W., Yang Y., Guo T.-Z., Sahbaie P., Shi X.-Y., Guang Q., Kingery W.S., Herzenberg L.A., Clark J.D. (2021). IL-6 signaling mediates the germinal center response, IgM production and nociceptive sensitization in male mice after tibia fracture. Brain Behav. Immun..

[8] Zhao Y., Zhang H., Li N., Li J., Zhang L. (2022). Chronic Pain after Bone Fracture: Current Insights into Molecular Mechanisms and Therapeutic Strategies. Brain Sci..

[9] Chen G., Zhang Y.Q., Qadri Y.J., Serhan C.N., Ji R.R. (2018). Microglia in pain: Detrimental and protective roles in pathogenesis and res-olution of pain. Neuron.

[10] Ji R.-R., Nackley A., Huh B.Y., Terrando N., Maixner D.W. (2018). Neuroinflammation and Central Sensitization in Chronic and Widespread Pain. Anesthesiology.

[11] Qiang Z., Yu W. (2019). Chemokine CCL7 regulates spinal phosphorylation of GluA1-containing AMPA receptor via interleukin-18 in remifentanil-induced hyperalgesia in rats. Neurosci. Lett..

[12] Gong G., Hu L., Qin F., Yin L., Yi X., Yuan L., Wu W. (2016). Spinal WNT pathway contributes to remifentanil induced hyperalgesia through regulating fractalkine and CX3CR1 in rats. Neurosci. Lett..

[13] Sessler K., Blechschmidt V., Hoheisel U., Mense S., Schirmer L., Treede R.-D. (2021). Spinal cord fractalkine (CX3CL1) signaling is critical for neuronal sensitization in experimental nonspecific, myofascial low back pain. J. Neurophysiol..

[14] Wang Z., Li L., Bian C., Yang L., Lv N., Zhang Y. (2018). Involvement of NF-κB and the CX3CR1 Signaling Network in Mechanical Al-lodynia Induced by Tetanic Sciatic Stimulation. Neurosci. Bull..

[15] Borghi S.M., Fattori V., Pinho-Ribeiro F.A., Domiciano T.P., Miranda-Sapla M.M., Zaninelli T.H., Casagrande R., Pinge-Filho P., Pa-vanelli W.R., Alves-Filho J.C. (2019). Contribution of spinal cord glial cells to L. amazonensis ex-perimental infection-induced pain in BALB/c mice. J. Neuroinflam..

[16] Cui W., Li Y., Wang Z.M., Song C., Yu Y., Wang G.M., Li J., Wang C., Zhang L. (2021). Spinal caspase-6 regulates AMPA receptor trafficking and dendritic spine plasticity through netrin-1 in postoperative pain after orthopedic surgery for tibial fracture in mice. Pain.

[17] Bindu S., Mazumder S., Bandyopadhyay U. (2020). Non-steroidal anti-inflammatory drugs (NSAIDs) and organ damage: A current perspective. Biochem. Pharm..

[18] Colvin L.A., Bull F., Hales T.G. (2019). Perioperative opioid analgesia—When is enough too much? A review of opioid-induced tolerance and hyperalgesia. Lancet.

[19] Ding Y., Brand E., Wang W., Zhao Z. (2022). Licorice: Resources, applications in ancient and modern times. J. Ethnopharmacol..

[20] Yang R., Yuan B.-C., Ma Y.-S., Zhou S., Liu Y. (2016). The anti-inflammatory activity of licorice, a widely used Chinese herb. Pharm. Biol..

[21] Li S., Wang Y., Wu M., Younis M.H., Olson A.P., Barnhart T.E., Engle J.W., Zhu X., Cai W. (2022). Spleen-Targeted Glabridin-Loaded Na-noparticles Regulate Polarization of Monocyte/Macrophage (Mo/Mφ) for the Treatment of Cerebral Ischemia-Reperfusion Injury. Adv. Mater..

[22] Park S.H., Kang J.S., Yoon Y.D., Lee K., Kim K.J., Lee K.H., Lee C.W., Moon E.Y., Han S.B., Kim B.H. (2010). Glabridin inhib-its lipopolysaccharide-induced activation of a microglial cell line, BV-2, by blocking NF-kappaB and AP-1. Phytother. Res..

[23] Parlar A., Arslan S.O., Çam S.A. (2020). Glabridin Alleviates Inflammation and Nociception in Rodents by Activating BKCa Channels and Reducing NO Levels. Biol. Pharm. Bull..

[24] Donnelly C.R., Jiang C., Andriessen A.S., Wang K., Wang Z., Ding H., Zhao J., Luo X., Lee M.S., Lei Y.L. (2021). STING controls nociception via type I interferon signalling in sensory neurons. Nature.

[25] Wang Y., Wang P., Liu C., Chen W., Wang P., Jiang L. (2022). Hydrogen-Rich Saline Attenuates Chronic Allodynia after Bone Fractures via Reducing Spinal CXCL1/CXCR2-Mediated Iron Accumulation in Mice. Brain Sci..

[26] Zhang L., Wang Z., Song C., Liu H., Li Y., Li J., Yu Y., Wang G., Cui W. (2021). Spinal NR2B phosphorylation at Tyr1472 regulates IRE (-) DMT1-mediated iron accumulation and spine morphogenesis via kalirin-7 in tibial fracture-associated postoperative pain af-ter orthopedic surgery in female mice. Reg. Anesth. Pain Med..

[27] Zhang L., Guo S., Zhao Q., Li Y., Song C., Wang C., Yu Y., Wang G. (2018). Spinal Protein Kinase Mζ Regulates α-Amino-3-hydroxy-5-methyl-4-isoxazolepropionic Acid Receptor Trafficking and Dendritic Spine Plasticity via Kalirin-7 in the Pathogenesis of Remifentanil-induced Postincisional Hyperalgesia in Rats. Anesthesiology.

[28] Bell R., Moreira V., Kalso E., Yli-Kauhaluoma J. (2021). Liquorice for pain?. Ther. Adv. Psychopharmacol..

[29] Wang C., Xu R., Wang X., Li Q., Li Y., Jiao Y., Zhao Q., Guo S., Su L., Yu Y. (2020). Spinal CCL1/CCR8 regulates phosphorylation of GluA1-containing AMPA receptor in postoperative pain after tibial fracture and orthopedic surgery in mice. Neurosci. Res..

[30] Zhang L., Li N., Zhang H., Wang Y., Gao T., Zhao Y., Wang G., Yu Y., Wang C., Li Y. (2022). Artesunate therapy alleviates frac-ture-associated chronic pain after orthopedic surgery by suppressing CCL21-dependent TREM2/DAP12 inflammatory sig-naling in mice. Front. Pharmacol..

[31] Lu J., Luo C., Bali K.K., Xie R.G., Mains R.E., Eipper B.A., Kuner R. (2015). A role for Kalirin-7 in nociceptive sensitization via activi-ty-dependent modulation of spinal synapses. Nat. Commun..

[32] Stratton H.J., Khanna R. (2020). Sculpting Dendritic Spines during Initiation and Maintenance of Neuropathic Pain. J. Neurosci..

[33] Grace P.M., Gaudet A.D., Staikopoulos V., Maier S.F., Hutchinson M.R., Salvemini D., Watkins L.R. (2016). Nitroxidative signaling mecha-nisms in pathological pain. Trends Neurosci..

[34] Squillace S., Salvemini D. (2021). Nitroxidative stress in pain and opioid-induced adverse effects: Therapeutic opportunities. Pain.

[35] Bhatt S., Sharma A., Dogra A., Sharma P., Kumar A., Kotwal P., Bag S., Misra P., Singh G., Kumar A. (2022). Glabridin attenuates paracetamol-induced liver injury in mice via CYP2E1-mediated inhibition of oxidative stress. Drug Chem. Toxicol..

[36] Zhang J., Wu X., Zhong B., Liao Q., Wang X., Xie Y., He X. (2023). Review on the Diverse Biological Effects of Glabridin. Drug Des. Dev. Ther..

[37] Li T., Chen X., Zhang C., Zhang Y., Yao W. (2019). An update on reactive astrocytes in chronic pain. J. Neuroinflam..

[38] Liu X., Bae C., Gelman B.B., Chung J.M., Tang S.-J. (2022). A neuron-to-astrocyte Wnt5a signal governs astrogliosis during HIV-associated pain pathogenesis. Brain.

